# Practical application of PMA–qPCR assay for determination of viable cells of inter-species biofilm of *Candida albicans–Staphylococcus aureus*

**DOI:** 10.1093/biomethods/bpae081

**Published:** 2024-11-18

**Authors:** Samuel Kendra, Jarmila Czucz Varga, Barbora Gaálová-Radochová, Helena Bujdáková

**Affiliations:** Faculty of Natural Sciences, Department of Microbiology and Virology, Comenius University in Bratislava, Bratislava, 84215, Slovakia; Faculty of Natural Sciences, Department of Microbiology and Virology, Comenius University in Bratislava, Bratislava, 84215, Slovakia; Faculty of Natural Sciences, Department of Microbiology and Virology, Comenius University in Bratislava, Bratislava, 84215, Slovakia; Faculty of Natural Sciences, Department of Microbiology and Virology, Comenius University in Bratislava, Bratislava, 84215, Slovakia

**Keywords:** PMA–qPCR, *Candida albicans*, *Staphylococcus aureus*, mixed biofilm, photodynamic inactivation

## Abstract

Determining the number of viable cells by calculating colony-forming units is time-consuming. The evaluation of mixed biofilms consisting of different species is particularly problematic. Therefore, the aim of this study was to optimize a molecular method—propidium monoazide quantitative polymerase chain reaction (PMA–qPCR)—for accurate and consistent differentiation between living and dead cells. In the practical experimental example, the number of genome copies representing living cells was determined in a mixed biofilm of *Candida albicans*–*Staphylococcus aureus* inhibited by photodynamic inactivation. Optimal conditions such as PMA concentration and the duration of light exposure, the optimization of DNA isolation from the mixed biofilm and standardization of PMA–qPCR parameters were tested prior to the main experiment. The genome copy number was calculated based on the known amount of genomic DNA in the qPCR and the genome size of the respective microorganism. The results showed that photodynamic inactivation in the presence of 1 mM methylene blue decreased the total genome copy number from 1.65 × 10^8^ to 3.19 × 10^7^, and from 4.39 × 10^7^ to 1.91 × 10^7^ for *S. aureus* and *C. albicans* (*P *<* *0.01), respectively. The main disadvantage is the overestimation of the number of living cells represented by genome copy numbers. Such cells are unable to reproduce and grow (no vitality) and are continuously dying. On the other hand, PMA–qPCR determines the copy numbers of all microbial species, including a mix of eukaryotic yeasts and prokaryotic bacteria in a biofilm in one step, which is a great advantage.

## Introduction

The determination of cell viability is the most common approach used for testing the effects of various factors on cell survival. It is defined as the number of live cells in a population. Samples are serially diluted according to Koch’s protocol and plated to solid media. The living cells forming colonies are calculated and then estimated per milliliter (colony forming unit—CFU/ml) [1, 2]. Despite the simplicity of this method, it is very time-consuming, and human influence has a significant impact, as good pipetting practices are of utmost importance. In biofilm testing, extra steps, such as sonication or enzymatic disruption of the extracellular polymeric substance (EPS) need to be used, which can also affect the viability of biofilm cells [3]. Additionally, in a multi-species biofilm, there are specific recommended media for discriminating between bacteria and fungi or between bacteria alone. The particular limitation of CFU counting is underestimating the real number of viable cells because of their limited vitality, leading to an inability to grow and form colonies on solid media. There is evidence that after the treatment of cultivable bacteria such as *Staphylococcus aureus*, *Escherichia coli*, or *Pseudomonas aeruginosa*, stressful conditions can keep them in a viable, but non-culturable (VBNC) state [4–7]. These forms of bacteria can be detectable by other more sensitive methods [5, 8].

In recent years, some advanced methods such as flow cytometry or fluorescence *in situ* hybridization have been utilized, providing information on cell survival with high accuracy [9–12]. However, both techniques require highly trained personnel and expensive equipment, which are limitations for daily laboratory practice. In contrast to this, a molecular technique based on quantification of the number of genome copies can be performed easily in a common biological laboratory. The principle of propidium monoazide (PMA) quantitative polymerase chain reaction (qPCR) is based on the determination of viable cells using a photosensitizer (PS) that is only able to enter membrane-compromised cells and then to intercalate into dsDNA. After exposure to visible light, PMA forms a covalent linkage with the dsDNA, resulting in permanent DNA modification that leads to the inhibition of PCR amplification [13, 14]. In addition to PMA, in the case of the yeast *Candida albicans*, ethidium bromide monoazide is also a suitable alternative, but PMA has been shown to be less cytotoxic and less permeable to intact cells to bacteria [14–17]. Additionally, the advantage is that PMA also blocks extracellular dsDNA, which is commonly present in the EPS of biofilms [13, 18]. These complexes are removed during DNA isolation [19, 18–20]. The final evaluation can be expressed as either the percentage of viable cells or the absolute number of viable cells in a sample [21–23]. However, quantification in terms of the number of genome copies (per reaction, volume) is more accurate. The copy number needs to be calculated based on the known amount of DNA in the qPCR reaction mixture and on the genome size of the respective microorganism [21, 24].

To date, viability PMA–qPCR has been most widely used for various microorganisms [23, 25–27]. However, this method has not been applied very often for assessing the viability of microbial biofilms. A great deal of experience has been obtained with *Listeria monocytogenes* biofilms treated with different compounds, as these bacteria often manifest the VBNC phenotype [28, 29]. PMA–qPCR was also used for the quantitation of viable *E. coli* O157: H7 in a multi-species biofilm with *Acinetobacter* and *Bacillus* spp. [30].

The objective of this study was to optimize and describe in detail the use of a PMA–qPCR assay on a mixed biofilm formed by prokaryotic bacteria of *S. aureus* and eukaryotic yeast *C. albicans* with a practical application—testing the effectiveness of photodynamic inactivation (PDI) on this mixed biofilm. PDI is a new alternative approach for fighting resistant and biofilm-forming microorganisms with promising results [31–34]. The manuscript also showed the advantages and limitations of PMA–qPCR in a study of mixed biofilms.

## Materials and methods

### Strains, culture preparation, and optimization of PCR conditions

For the dual biofilm, the yeast *C. albicans* SC 5314 [35] and the bacterium *S. aureus* CCM 3953 (corresponding to ATCC 25923, Czech Collection of Microorganisms, Brno, Czech Republic) were used. For overnight cultivation, Mueller Hinton broth (MHB, Biolife, Milan, Italy) or yeast extract–peptone–dextrose (YPD, 2% glucose, 1% yeast extract, 2% peptone; w/v; all from Biolife, Milan, Italy) were used.

One loopful of cells from the respective strain was cultivated in 20 ml of MHB or YPD in an orbital shaker (Multitrone Standard, Bottmingen-Basel, Switzerland) at 150  RPM for up to 16 h at 37°C and 28°C for *S. aureus* and *C. albicans*, respectively. Before each experiment, *S. aureus* was prepared from overnight culture to a optical density (OD) of OD_570_ = 0.05 (Dynex, MRX-TC Revelation, Dynex Technologies, USA). Then, bacteria were cultivated to the exponential phase of growth (OD_570_ = 0.5), corresponding to ≈1 × 10^8^ cells/ml. An overnight culture of *C. albicans* was washed twice with phosphate-buffered saline buffer (PBS) (Sigma Aldrich, Darmstadt, Germany), and the cells were pelleted by centrifugation at 5000*g* for 5 min. The pellet of *C. albicans* was resuspended in 10 ml of PBS. Cell density was counted in a Bürker chamber (Paul Marienfeld GmbH & Co., Lauda-Königshofen, Germany) and estimated to a final density of 4 × 10^6^ cells/ml in MHB supplemented with 2% glucose. Microorganisms prepared in this way were used in all experiments.

Table 1 summarizes the primers for PCR; the primers for the *nuc* gene were provided in a PMA Real-Time PCR Bacterial Viability Kit—*Staphylococcus aureus* (Biotium, Fremont, USA, RRID: SCR_013538) [36] and were previously described by Fang and Hedin [37]. Primers for the housekeeping gene *ACT1* were designed by Dr Imrich Hikkel (Faculty of Natural Sciences, Comenius University in Bratislava, Slovakia). Both primers were synthesized by Metabion (Planneg, Germany).

**Table 1.** Oligonucleotide sequences for specific detection of *S. aureus* CCM 3953 and *C. albicans* SC 5314 by qPCR.

Strain	Gene	Oligonucleotide sequence	**Tm,** °C	Product size, bp
*Staphylococcus aureus* CCM 3953	*nuc*	For: 5’-GCGATTGATGGTGATACGGTT-3″ Rev: 5’-AGCCAAGCCTTGACGAACTAAAGC-3″	59 64	279
*Candida albicans* SC 5314	*ACT1*	For: 5’-CTCTTCTGGTAGAACCACCGGTAT-3″ Rev: 5’-TAAAGAGAAACCAGCGTAAATTGGA-3″	65 61	85

The optimal melting temperature for both sets of primers was determined by gradient PCR (C1000 Touch PCR thermal cycler, BIORAD, Hercules, USA). The effectiveness of the PCR at the final annealing temperature of 55°C was evaluated by gel electrophoresis (Supplementary data, Figs S1 and S2).

### Preparation of single and mixed biofilms, treatment of biofilm cells with PMA, and isolation of genomic DNA

For single biofilms, 200 μl of cell suspensions of *C. albicans* or *S. aureus*, prepared as described previously, were added into a 96-well microtiter TC plate (Sardstedt AG & Co, Germany) in MHB supplemented with 2% glucose and incubated at 37°C for 24 h.

**Figure 1.**
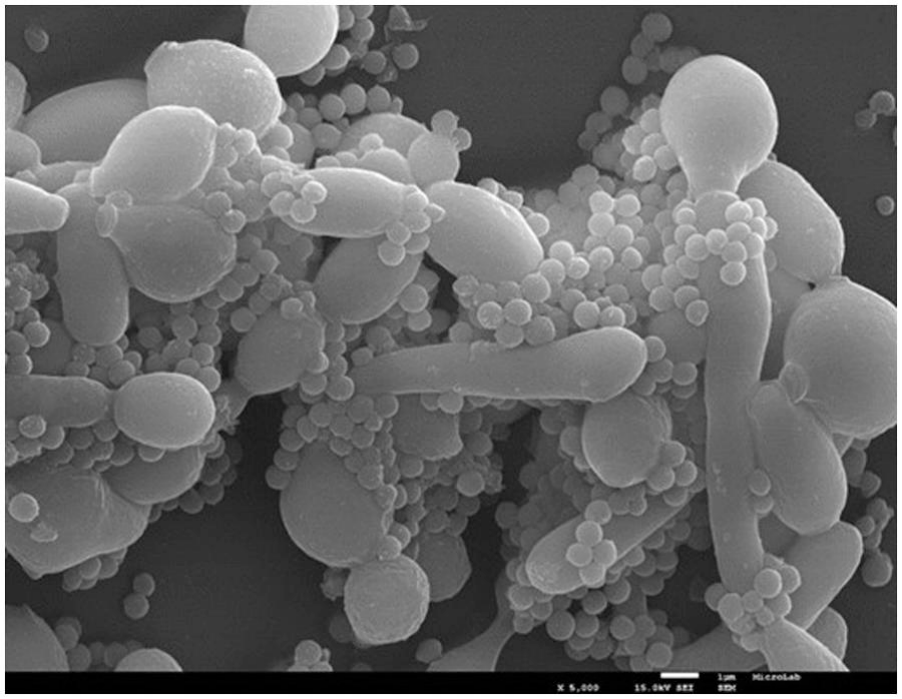
SEM micrograph of 48-h mixed biofilm of *C. albicans–S. aureus*. The scale bars are 1 µm.

The mixed biofilm of *C. albicans–S. aureus* (Fig. 1) was prepared as follows: briefly, 200 μl of *C. albicans* suspension in MHB with 2% glucose (prepared as previously described) was added into a 96-well microtiter plate and incubated statically at 37°C (Thermostatic Cabinet, Lovibond, Germany). After 24 h, the medium was carefully aspirated, and 50 μl of *S. aureus* suspension (prepared as described previously) was added. Wells were then filled with 150 µl of MHB containing 2% glucose. Cultivation continued for another 24 h. For visualization, the mixed biofilm was fixed with 4% paraformaldehyde (Sigma-Aldrich, Steinheim, Germany) in PBS and incubated for 1 h in the dark at room temperature (RT). The fixative was removed, and the samples were washed twice in PBS for 10 min. Samples were post-fixated with 1% osmium tetroxide (EMS, Hatfield, PA, USA) in PBS for 1 h in the dark at RT. Then, the samples were washed twice in PBS and deionized water for 10 min each at RT. Dehydration was performed using a serial dilution of ethanol: 25%, 50%, 70%, and 95% (Sigma-Aldrich, Steinheim, Germany), with each step for 15 min in the dark at RT. Finally, 100% ethanol was added for 30 min, and this step was repeated one more time. Samples were allowed to dry at RT in a chamber and were then assembled on double-sided carbon tape.

Sputter-coated samples with carbon (20 nm) using a Sputter Coater QISOT ES (Quorum Technologies, Lewes, UK) were mounted on the scanning electron microscopy (SEM) sample holder with carbon tape and analyzed under an electron microscope, JSM-7100F (JEOL, Tokyo, Japan).

For each microorganism, samples for the live control biofilm (positive control) and dead control biofilm (negative control) and both with and without subsequent PMA treatment were prepared. The dead control biofilm samples were prepared prior to PMA treatment by inactivating microorganisms at 100°C (Mini Cooling/Heating Dry Bath Incubator, Major Science CO., Ltd, Taoyuan City, Taiwan) for 20 min. Zero viability of microorganisms after heating was confirmed by parallel plating onto MHA with fluconazole (4 µg/ml, Zentiva, Prague, Czech Republic) for selection of *S. aureus* and YPD with gentamicin (4 µg/ml, Applichem, Darmstadt, Germany) for growth of *C. albicans*.

After forming both single and mixed biofilms, the medium was removed from the wells and 100 µl of MHB without glucose was added, biofilms were scraped off from four wells per sample, pooled (total 400 µl), and transferred into a 1.5 ml transparent Eppendorf tube (Sarstedt, Nümbrecht, Germany). Prior to PMA treatment, the PMA dye was diluted in water. To find an optimal concentration of PMA that does not affect the viability of live cells of both microorganisms, different concentrations of PMA were tested (10, 25, 28, 30, 40, 50 µM) (data not shown). Based on these results, 25 µM PMA was selected as the optimal concentration for further experiments (Supplementary data, Fig. S3). Afterward, all samples were incubated in the dark for 10 min at RT on a rocker (Multi-Vortex V-32, Biosan, Riga, Latvia). Subsequently, all samples were exposed to light with 465–475 nm emission using a PMA-Lite^TM^ LED Photolysis Device, specifically designed for photoactivation of PMA (Biotium, Fremont, USA), for 15 min. During irradiation, the tubes were covered with aluminum foil on top of the device to reflect the light downward. After irradiation, the cells were pelleted by centrifuging at 5000*g* for 10 min (High-Speed Microliter Centrifuge Frontier™ 5515R, Ohaus Europe GmbH, Nänikon, Switzerland), the supernatant was removed, and 750 µl of DNA/RNA Shield^TM^ from a ZymoBIOMICS^TM^ DNA/RNA Miniprep Kit (Zymo Research, Orange, USA, RRID: SCR_008968) was added to each sample. Samples were stored at −20°C until used, but at most for 1 week.

The extraction of genomic DNA (gDNA) from biofilm samples was performed with a ZymoBIOMICS^TM^ DNA/RNA Miniprep Kit according to a protocol of provider that was slightly modified. The 200 μl of Yeast lysis buffer containing 1 M sorbitol (AppliChem GmbH, Darmstadt, Germany), 0.1 M EDTA, pH 7.4 (Thermo Fisher Scientific, Waltham, USA), 0.1% β-mercaptoethanol (AppliChem GmbH, Darmstadt, Germany), and 200 U of lyticase (Sigma Aldrich, Darmstadt, Germany) were added to each sample in 750 µl of DNA/RNA Shield^TM^. The samples were vortexed for 1 min and incubated at 30°C for 60 min. In the next step, the samples were transferred to a ZR BashingBead Lysis Tube 0.1 and 0.5 mm (part of the kit), and all samples were vortexed for 60 min at 2550 RPM (Vortex—Genie 2, Scientific Industries, Bohemia, USA). Subsequently, the lysates were centrifuged for 1 min at 16 000*g* and the supernatant was removed. Then the isolation of total gDNA from the supernatant continued using ZymoBIOMICS^TM^ DNA/RNA Miniprep Kit according to the protocol of kit’s provider. The concentration of isolated gDNA was measured (NanoDrop Technologies, Wilmington, USA), and samples of gDNA were stored at −20°C until used. Two independent isolations were done, and qPCR was repeated for minimum two times with three parallel wells for each isolated DNA.

### PDI of mixed biofilm in the presence of methylene blue

The procedure and conditions for the preparation of a 48-h mixed biofilm of *C. albicans* and *S. aureus* have been described above. Before using PMA, some biofilms were treated with 1 mM of PS methylene blue (MB) (Loba Biotech GmbH, Fischamend, Austria) according to the protocol of Černáková *et al*. [38]. Briefly, after removing the medium from a 48-h biofilm, 100 µl of 1 mM MB diluted in PBS was pipetted into the wells, followed by 1.5-h incubation at RT in the dark. Control wells with biofilm were filled with 100 µl of PBS. After 1.5-h incubation, MB and PBS were removed. Samples of biofilms with MB incubation assigned to PDI were irradiated with a red laser (λ = 660 nm, 190 mW cm^−2^) for 5 min, corresponding to a fluence of 57 J cm^−2^. Biofilms with MB incubation without irradiation, as well as the control biofilm with PBS (without MB), were kept in the dark. Additionally, the effect of light was tested on the control sample. At least four parallel wells were prepared for each set of treatment conditions. After PDI, 100 µl of MHB without glucose was added to each well, four wells were pooled, and the protocol for PMA treatment was followed as described in the previous paragraph. The cells were pelleted by centrifugation at 5000*g* for 10 min, supernatants were removed, and 750 µl of DNA/RNA Shield^TM^ was added to PMA-treated biofilm cells. The experiment also included live and dead control biofilm samples, both with or without PMA treatment, as described in the previous section. Samples were stored at −20°C until used, but at most for 1 week. Afterward, gDNA was isolated from all samples according to the procedure described in ‘Preparation of single and mixed biofilms, treatment of biofilm cells with PMA, and isolation of genomic gDNA’ section.

### PMA–qPCR, preparation of standard curves with gDNA, and calculation of number of genome copies

For the preparation of standard curves, single biofilms of *S. aureus* and *C. albicans* were prepared without PMA treatment according to the procedure described in ‘Preparation of single and mixed biofilms, treatment of biofilm cells with PMA, and isolation of genomic gDNA’ section. Extractions of gDNA from single biofilms of *S. aureus* and *C. albicans* were done as described in ‘Preparation of single and mixed biofilms, treatment of biofilm cells with PMA, and isolation of genomic gDNA’ section. Subsequent qPCR was carried out in a 96-well PCR plate (Natural 96 Well 900098, Deltalab, Barcelona, Spain) with the respective primers and DNA templates. Briefly, the total volume of reaction mixture was 20 µl; for the *nuc* gene, the mixture contained 10 µl of 2× Forget-Me-Not^TM^ Master Mix, 2 µl of 5 µM *nuc* Primer mix, 3 µl of ROX (1:100) (all components from Biotium, USA) [36], 3 µl of nuclease-free water (Canvax™ UltraPure Water, Canvax, Valladolid, Spain), and 2 µl of *S. aureus* gDNA. The reaction mixture for the *ACT1* gene consisted of 4 µl of 5× HOT FIREPol^®^ EvaGreen^®^ qPCR Mix Plus (ROX) (Solis BioDyne, Tartu, Estonia), 0.5 µl of *ACT1* Forward Primer and 0.5 µl of *ACT1* Reverse Primer (Metabion, Planneg, Germany), 13 µl of nuclease-free water and 2 µl of *C. albicans* gDNA. Each sample was pipetted in three parallel wells. The qPCR was run (Stratagene Mx3000P Real-Time PCR System, RRID: SCR_020526) according to the conditions listed in Table 2. The negative control contained nuclease-free water instead of gDNA. In qPCR for samples with PDI, the optimal dilution of gDNA was verified based on Ct values, and finally estimated to be 100-fold. PMA-qPCR was run in one plate but as single-plex qPCR. After amplification, a melting curve was run to ensure the absence of primer dimers and to confirm the annealing temperature during the cycle (Supplementary data Fig. S4).

**Table 2.** Parameters for PMA-qPCR with gDNA of *C. albicans* SC 5314 and *S. aureus* CCM 3953.

Step	Temperature, °C	Time (min)	Cycles
Initial denaturation	95	12:00	1×
Denaturation	95	00:15	40×
Annealing and extension	55	01:00	
Complementary phase	95	00:15	1×
	55	00:15	
	95	00:15	

The concentration of isolated gDNA was normalized to 10 ng/µl, which was the stock solution of gDNA. Considering the volumes of gDNA added to the reaction mixture for qPCR, the total amount of gDNA per reaction was set to 20 ng. For the generation of standard and amplification curves, six serial dilutions (from 20 ng to 0.2 pg) of gDNA in nuclease-free water were run. Standard curves were generated with MxPro QPCR software (Agilent Technologies, Inc., Santa Clara, USA, RRID: SCR_016375). Based on the genome size of *S. aureus* containing ≈2800 kb [39], one copy of *S. aureus* represents ≈3 fg. The diploid genome of *C. albicans* is ≈29 400 kb [40], which means that genome copy of *C. albicans* represents ≈32 fg. The molecular weight of genomes was calculated according to the formula [41]:

$$m=\left(n\right)\left(1.096{\times 10}^{-21}\;\frac{g}{\text{bp}}\right)$$

where *n* = genome size (bp); *m* = mass

After calculation, 20 ng of gDNA used for standard curves correspond to 6.67 × 10^6^ and 6.25 × 10^5^ genome copies per reaction for *S. aureus* and *C. albicans*, respectively.

### Determination of viable cells from PMA–qPCR

In preliminary experiments, serial gDNA dilutions of all samples of single and mixed biofilms (live and dead biofilm cells, both with and without PMA) were run in PMA–qPCR.

To determine whether PMA only inhibited the gDNA amplification of dead cells, the delta Ct (dCt) was calculated for positive as well as negative control biofilm cells [36]:

$${\text{dCt}}_{\text{live}\;\text{cells}}={\text{Ct}}_{\left(\text{live}\;\text{with}\;\text{PMA}\right)}-{\text{Ct}}_{\left(\text{live}\;\text{without}\;\text{PMA}\right)}$$

 

$${\text{dCt}}_{\text{dead}\;\text{cells}}={\text{Ct}}_{\left(\text{dead}\;\text{with}\;\text{PMA}\right)}-{\text{Ct}}_{\left(\text{dead}\;\text{without}\;\text{PMA}\right)}$$

The expected difference in dCt between the live biofilm cells (positive control) with PMA and without PMA should be 0 (±1 ), and for dead cells (negative control), it should be more than four cycles. The calculation of the number of genome copies in an unknown sample per reaction was based on the obtained dCt values for each sample using the software MxPro QPCR utilizing the standard curves of individual microorganisms.

### Statistical analysis

Data were evaluated for normality using the Shapiro–Wilk test (software Graph Pad Prism 9, San Diego, USA, RRID: SCR_002798) and for homogeneity of variances by one-way ANOVA (Analysis of variance) on the absolute residuals (corresponding to Levene’s test) (Excel, Microsoft 365, v.2402). Comparison between tested groups was done by Wilcoxon signed-rank test, one-tailed (Graph Pad Prism 9). Differences were considered statistically significant at different *P*-values: *P <* *0.05, **P <* *0.01.

## Results and discussion

### Optimization of PMA–qPCR parameters

The definition of dead cells in microorganisms is associated with the functionality of the cell membrane. If it is damaged in some way and does not carry out its functions, the cells die. PMA–qPCR can distinguish between living and dead cells in a sample based on the selective binding of the dye to the DNA of dead cells [14, 23]. Subsequent specific identification of DNAs originating from a dual-species biofilm relies on targeting specific gene sequences with oligonucleotide primers [37, 42]. The aim was to optimize and launch the PMA–qPCR according to such parameters, to achieve selective amplification of the target DNA during the same run. This can eliminate discrepancies that can occur when the amplification of each specific DNA is carried out in a separate run. Prior to all experiments, the optimal melting temperature of both sets of primers was determined using gradient PCR. It was set to 55°C. This temperature was also used in PMA–qPCR. Based on the dissociation curve obtained from qPCR with gDNA of both strains, the presence of a specific amplicon in the reaction mixture was confirmed (Supplementary data, Fig. S4).

### Optimization of efficient concentration of PMA dye, duration of incubation, and photoactivation

**Figure 2.**
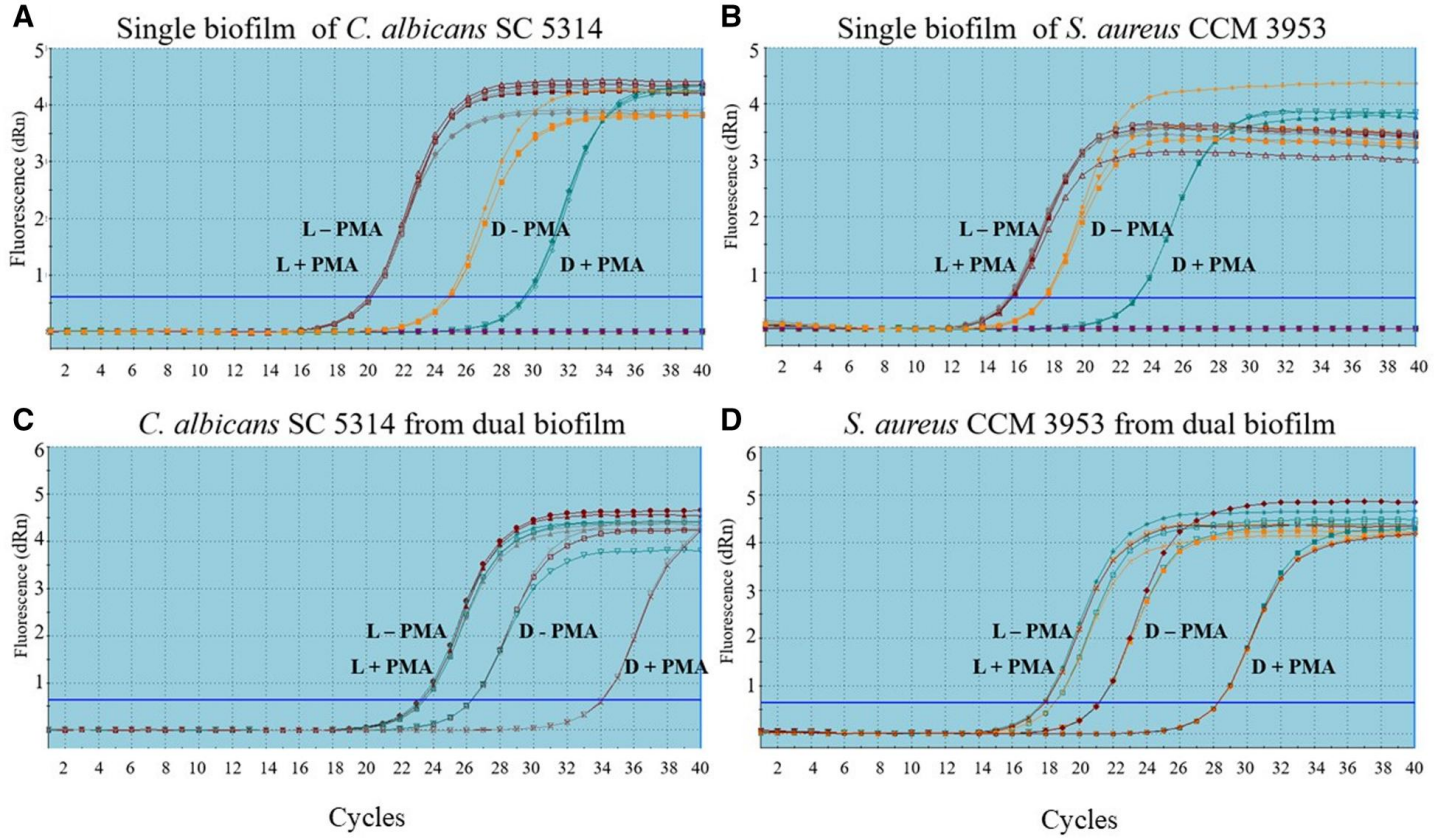
Amplification curves for gDNA of live and dead biofilm cells with and without 25 µM PMA. DNA originated from single biofilm of *C. albicans* (A) and *S. aureus* (B), as well as from *C. albicans* and *S. aureus* dual biofilms (C and D, respectively). PMA–qPCR was performed using gDNA of each strain with the respective primers. L + PMA = live cell control with PMA; L − PMA = live cell control without PMA; D + PMA = dead cell control with PMA; D − PMA = dead cell control without PMA.

The efficiency of PMA–qPCR can be affected by three methodological factors—the concentration of PMA, duration of incubation, and period of photoactivation [43]. An optimal PMA concentration had to be determined to avoid the interaction of the dye with the DNA of viable cells. In this study, different concentrations of PMA were tested on biofilm viability. To determine whether PMA only inhibited the gDNA amplification of dead cells, dCt was calculated for a positive as well as for a negative control, as described in ‘Determination of viable cells from PMA–qPCR’ section. As was previously mentioned, PMA selectively penetrates through damaged membranes and blocks DNA amplification [18, 20]. According to preliminary tests (Supplementary data, Fig. S5), 25 µM PMA was selected as optimal for PMA–qPCR, as documented in Fig. 2. Amplification curves of live cell controls with 25 µM PMA and without PMA almost overlapped. Differences in dCt were calculated as described in ‘Determination of viable cells from PMA–qPCR’ section (single biofilms: dCt_live_  _*C. albicans*_ = 0.03; dCt_live_  _*S. aureus*_ = 0.18; mixed biofilms: dCt_live_  _*C. albicans*_ = 0.39; dCt_live_  _*S. aureus*_ = 0.75). On the other hand, dCt values for the dead cell controls were higher than four cycles (single biofilms: dCt_dead_  _*C. albicans*_ = 4.59; dCt_dead_  _*S. aureus*_ = 5.48; mixed biofilms: dCt_dead_  _*C. albicans*_ = 4.005; dCt_dead_  _*S. aureus*_ = 6.62).

Furthermore, PMA–qPCR requires a photoactivation step for the dye to covalently bind to the DNA of dead cells [13, 14]. In general, the brighter the light is the more efficient the photolysis. Previously, PMA–qPCR assays employed visible light using high-power halogen lamps. However, this light heated the samples, which negatively affected the assay. Although halogen lamps are still sometimes used in viability PCR assays [15, 44–46], LED-based light sources are significantly more common and more efficient for performing this function [47]. They are less time-consuming to operate, do not overheat the samples, and offer easier handling and the simultaneous irradiation of multiple samples. A PMA-Lite™ LED Photolysis Device with a self-cooling system and with emission wavelengths from 465 to 475 nm has advantages over a halogen lamp. The irradiation period was set to 15 min with an intensity of 60 W. These irradiation times were different for *Legionella pneumophila* (2; 5; 15 min), where authors used different halogen lamps with an intensity of 500 W [15, 24, 25]. The incubation time of the sample with PMA, prior to irradiation, was set to 10 min. For *L. pneumophila*, publications indicate an incubation of 10–30 min [15, 24, 25]. Additionally, it is important to consider that eukaryotic yeast cells have a nuclear membrane and specific thick cell walls that can hinder efficient penetration of PMA. Therefore, optimization of PMA concentration and exposure time are essential.

### Generation of standard curves with gDNA for use in PMA–qPCR

**Figure 3.**
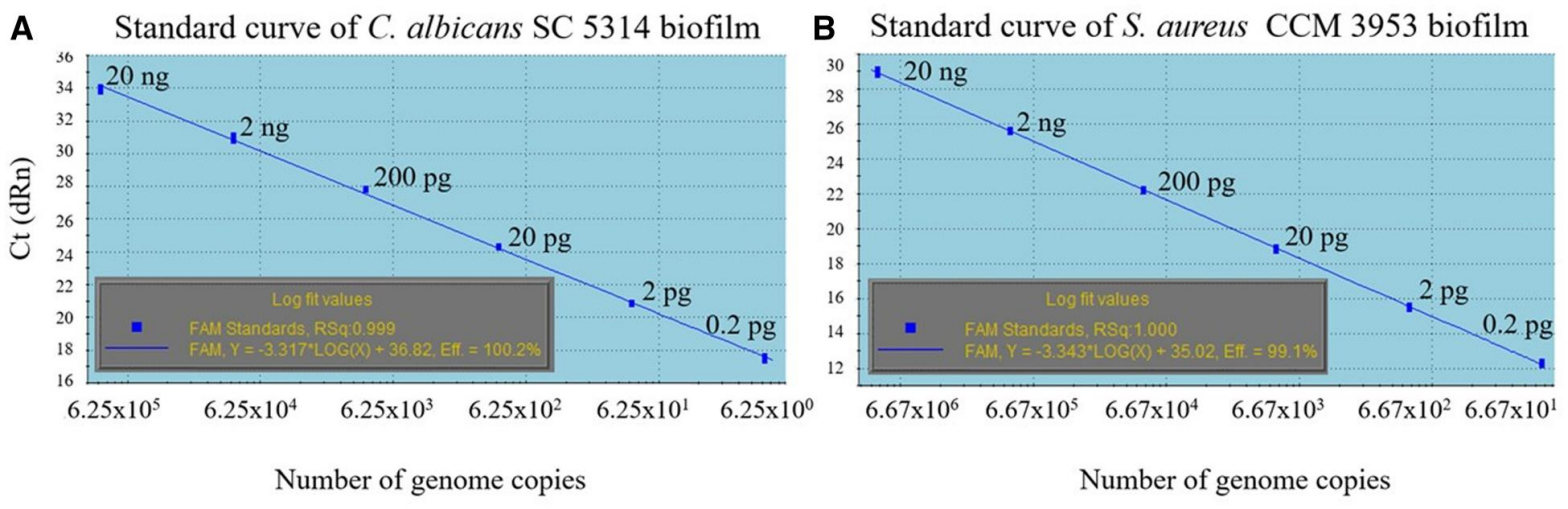
Standard curves from single biofilm for PMA–qPCR of *C. albicans ACT1* gene (A) and *S. aureus nuc* gene (B).

For the application of the PMA–qPCR assay in a practical experiment, it was necessary to construct standard curves with different dilutions of *C. albicans* and *S. aureus* gDNA. Standard curves for *C. albicans* and *S. aureus* were generated based on the specific amplification of the *ACT1* and *nuc* genes (Fig. 3). Using the software MxPro QPCR, Ct values (*y*-axis) were assigned to the corresponding number of genome copies per PCR displayed on the *x*-axis. The achieved standard lines exhibited appropriate parameters, such as linearity, amplification effectivity, and the correlation coefficient *R*^2^. The copy number was calculated from the known amount of gDNA in the qPCR mixture and from the genome size of the respective microorganism. The calculated number of copies is not equivalent to the number of CFUs [21].

**Figure 4.**
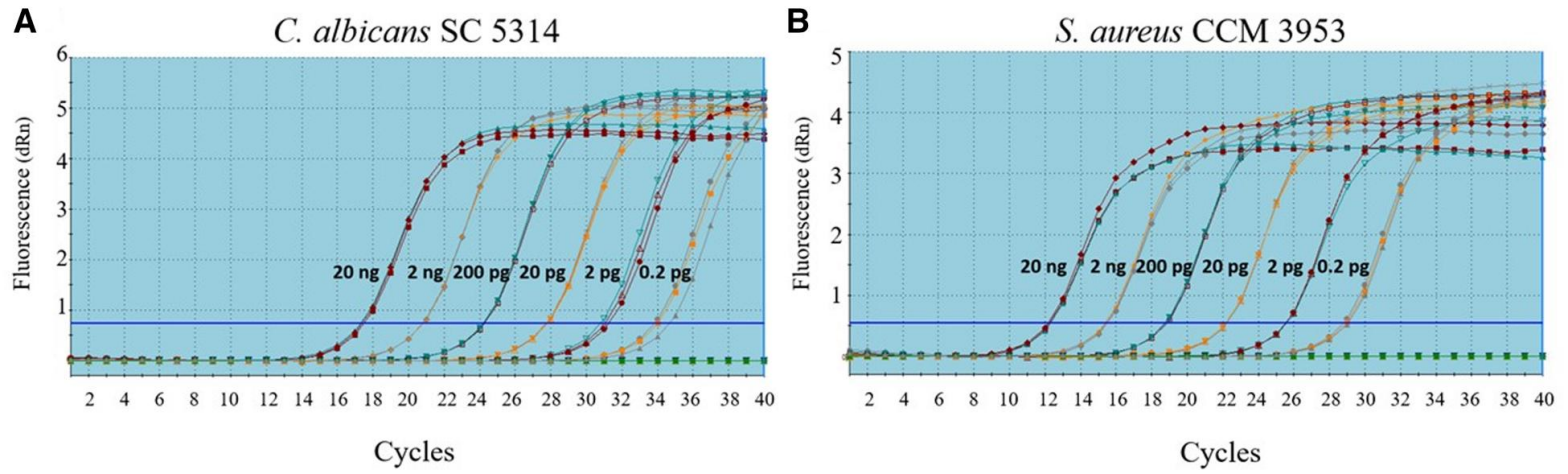
Amplification curves of six different DNA dilutions from single biofilms in qPCR with respective primers for *C. albicans* SC 5314 (A) and *S. aureus* CCM 3953 (B).

Amplification curves of six different DNA dilutions from single biofilms with the respective primers for *C. albicans* SC 5314 and *S. aureus* CCM 3953 are summarized in Fig. 4A and B, respectively. For the experiment with PDI, the concentration of gDNA was set to 20 ng for both single and mixed biofilms.

### Determination of the inhibition effect of PDI on *C. albicans*-*S. aureus* mixed biofilm by optimized PMA–qPCR assay

Among the three criteria (cultivability, metabolic activity, and membrane integrity) for viability assessments, membrane integrity is the most reliable criterion for distinguishing between live and dead cells [48, 49]. The PMA–qPCR method uses membrane integrity, but the main drawback of qPCR is its incomplete ability to distinguish viable and culturable cells from VBNC cells [17, 50]. VBNC cells are unable to form colonies, but remain alive and are able to resume their metabolic activity. This phenomenon is probably related to cellular integrity, which still allows some form of measurable cellular activity [6]. In addition, membrane integrity is not a measure of viability itself, but it more likely indicates how effective the particular treatments are at inflicting damage before cell death [51]. On the other hand, the PMA–qPCR could be used for quantification of microorganisms during infection, as already described by Sterzenbach *et al.* [52].

PMA–qPCR is not fully capable of capturing the current state of viable cells, unlike microscopy, flow cytometry, or the mapping of gene expression and protein synthesis [1, 6]. Soares *et al*. demonstrated that CFU/ml of *C. albicans* biofilm underestimated cells/ml, while qPCR using ethidium monoazide overestimated cells/ml. Additionally, they proved only poor agreement and lack of linear relationship between both methodologies and no association with transformation to hyphal form [53].

However, despite PMA–qPCR is unable to discriminate between viable and VBNC microbial cells, from the point of view of evaluating mixed biofilms, it seems to be a suitable method, as cultivation techniques are very time-consuming and reproducibility is strongly dependent on human factors.

Some other limitations of PMA–qPCR were described by Vondrakova *et al*. [51]. They suggested that the killing methods, and even species-specific differences, can affect PMA–qPCR efficacies, because some microbial species from the genus *Campylobacter* are resistant to PMA treatment (*C. lari*), and some are sensitive (*C. coli*). They proved that the efficiency of PMA–qPCR differed depending on the mechanism of cell inactivation and indicated that substances causing direct damage to bacterial cells in the form of non-thermal plasma generating reactive oxygen species (ROS) had the most pronounced effect on the cell membrane [51].

**Figure 5.**
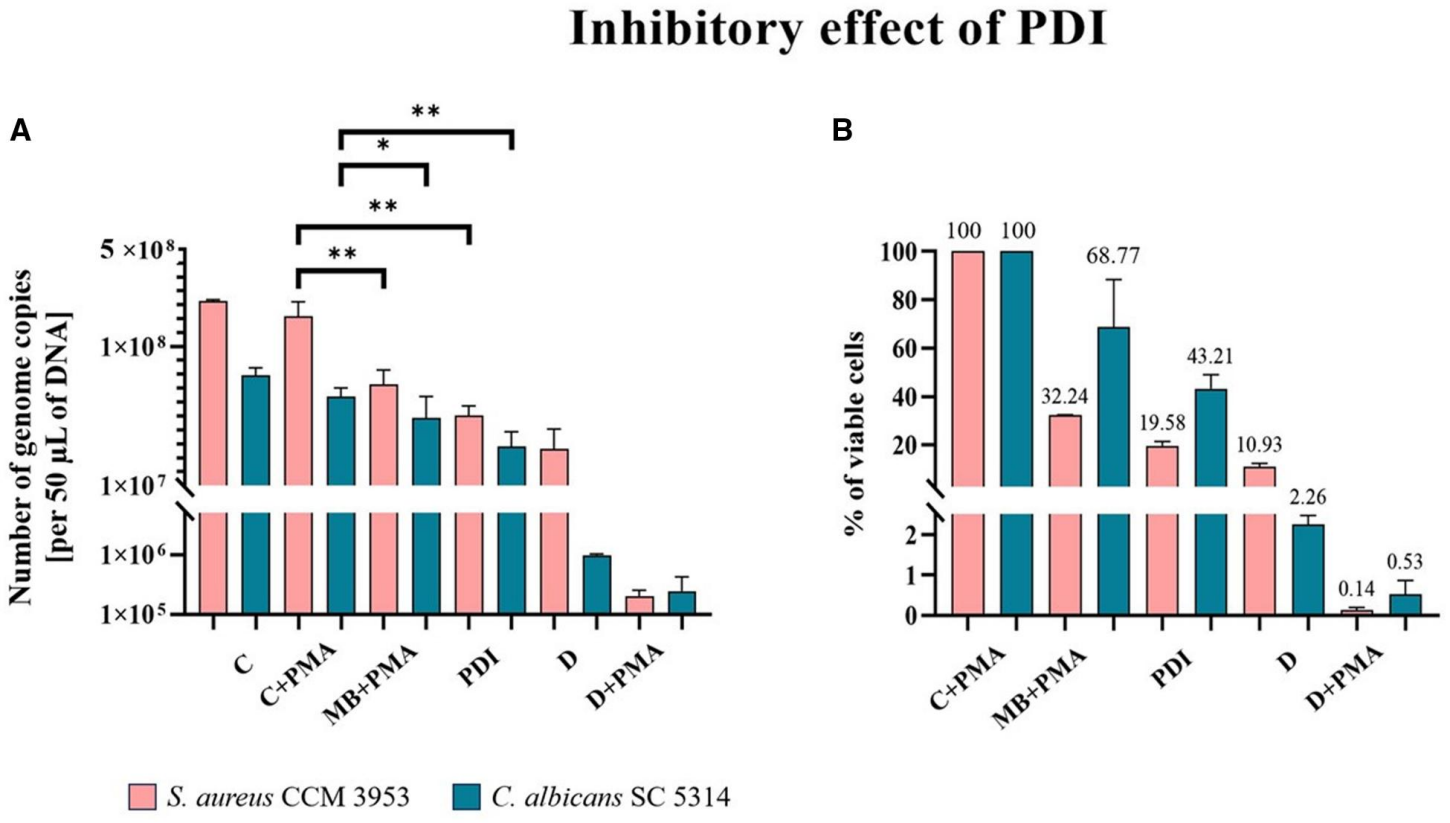
Determination of the inhibitory effect of PDI in the presence of MB on 48-h dual biofilm of *C. albicans* SC5314 and *S. aureus* CCM 3953 shown in total number of genome copies in 50 µl of DNA (A) and percentage of viable cells after PDI (B) using optimized PMA–qPCR method. C = live cells control without PMA and MB; C + PMA = live cells control with PMA; MB + PMA = control of toxicity of MB + PMA; PDI = cells with MB + PMA after PDI; D = dead cell controls without PMA; D + PMA = dead cell controls with PMA

In PDI, the killing of pathogenic microorganisms is achieved by applying a light source with a specific wavelength to activate the PS, which enters an excited state. The excited PS subsequently undergoes various photochemical processes, leading to the production of ROS, which cause irreversible damage to various molecules and cell structures, including the cell membrane [54, 55]. Therefore, PMA–qPCR was used to determine the efficacy of PDI on mixed biofilms of *C. albicans–S. aureus* in the presence of 1 mM MB. In PMA*–*qPCR, gDNA was used at a 100-fold dilution for every sample. The results summarized in Fig. 5 showed that PDI reduced the 48-h mixed biofilm, with different inhibitory effects on *C. albicans* and *S. aureus.*  Figure 5 (A) shows that PDI reduced the cell number represented by genome copies to 3.19 × 10^7^ for *S. aureus* (*P *<* *0.01) and 1.91 × 10^7^  *C. albicans* (*P *<* *0.01), respectively, compared to 1.65 × 10^8^ and 4.39 ×10^7^ estimated in the PMA-treated control samples. The inhibition effect of MB without irradiation was observed as well, as a relatively high concentration of MB was tested; 5.32 × 10^7^ for *S. aureus* (*P *<* *0.01) and 3.08 × 10^7^ for *C. albicans* (*P *<* *0.05). The control biofilm with PMA showed that part of the cell population spontaneously died compared to the control without PMA (from 2.13 × 10^8^ to 1.65 × 10^8^ and from 6.22 × 10^7^ to 4.39 × 10^7^) for *S. aureus* and *C. albicans*, respectively when the measurement was done in 48-h biofilms. Irradiation alone of control biofilm for 5 min did not show a significant inhibitory effect (Supplementary data, Fig. S6).

Figure 5 (B) shows cell survival as percentages. After PDI, the rate of survival was determined to be 19.58% and 43.21% for *S. aureus* and *C. albicans*, respectively, compared to the control biofilm treated with PMA. Dead cells served only as a negative control.

## Conclusions

The main idea of this manuscript was to find and optimize a method that would be suitable for various studies of mixed microbial biofilms. In this work, a mixed biofilm of the eukaryotic yeast *C. albicans* and the prokaryotic bacterium *S. aureus* was used in a practical application of testing the effectiveness of PDI. The conventional method of determining viability using CFU/ml needs microorganism-specific media to identify and calculate individual representatives in a biofilm, and so this method is very time-consuming. In addition, part of the cell population is not cultivable, because of the VBNC phenomenon. The above-mentioned weaknesses can lead to an underestimation of the final number of live cells. On the other hand, PMA–qPCR makes it possible to identify and at the same time quantify the individual microorganisms and number of live cells represented by the number of genomic units. PMA–qPCR also identifies microorganisms that manifest very low or no vitality associated with the so-called programmed cell death. In addition, the method is more demanding to optimize, which needs to be done for each tested microorganism alone, as well as for mixed biofilms. In summary, PMA–qPCR is a method that has the advantage of achieving “all-in-one” results, which enables a more accurate and faster analysis of multi-species biofilms.

## Supplementary Material

bpae081_Supplementary_Data

## Data Availability

Some raw data are available at https://doi.org/10.5281/zenodo.13754927. Other data are available on request.
