# Supplementary material for: Practical application of PMA–qPCR assay for determination of viable cells of inter-species biofilm of Candida albicans–Staphylococcus aureus
Source: Biol Methods Protoc. 2024 Nov 18;9(1):bpae081. doi: 10.1093/biomethods/bpae081 (PMC11631528; doi:10.1093/biomethods/bpae081)
Supplement: bpae081_Supplementary_Data [file bpae081_supplementary_data.zip › Supplementary data.docx]

**Supplementary Data**

**Practical application of PMA-qPCR assay for determination of viable cells of inter-species biofilm of *Candida albicans*-*Staphylococcus aureus***

**Samuel Kendra^1^, Jarmila Czucz Varga^1^, Barbora Gaálová-Radochová^1^, Helena Bujdáková^1*^**

^1^ Comenius University in Bratislava, Faculty of Natural Sciences, Department of Microbiology and Virology, Ilkovicova 6, 84215 Bratislava, Slovakia

*Corresponding author: prof. Helena Bujdakova, PhD. e-mail: [helena.bujdakova@uniba.sk](mailto:helena.bujdakova@uniba.sk)

*1.1 Gradient PCR and gel electrophoresis*

To verify the primers for PMA-qPCR, cycling conditions and the effect of PMA dye on DNA amplification, standard PCR was run with respective primers and gDNAs in a 0.2 mL Eppendorf tubes. *ACT1* primers were synthetized by Metabion (Planneg, Germany). *Nuc* primers were part of the kit PMA Real-Time PCR Bacterial Viability Kit *Staphylococcus aureus* (Biotium, Fremont, USA, RRID:SCR_013538). The sequences of the oligonucleotide primers, their melting temperatures and the expected size of the PCR products are listed in Table 1 (the main article).

The total volume of a reaction mixture was 20 µL and consisted of 4 µL 5 x FIREPol® Master Mix (Solis BioDyne, Tartu, Estonia), 0.5 µL of *ACT1* Forward primer and 0.5 µL of *ACT1* Reverse primer or 1 µL of *nuc* Primer Mix, 1 µL of respective template DNA, and 14 µL of nuclease-free water. The PCR was carried out in an iCycler Thermal Cycler (Bio-RaD, Hercules, California) according to the conditions listed in Table S1. A negative control containing nuclease-free water instead of gDNA was also included.

**Tab. S1:** PCR cycling parameters.

| **Step** | **Temperature** | **Duration (min)** | **Cycles** |
| --- | --- | --- | --- |
| Initial denaturation | 95 °C | 15:00 | 1x |
| Denaturation | 95 °C | 00:20 | 30x |
| Annealing (extension) | 55 °C* | 00:30 |  |
| Polymerization | 72 °C | 01:00 |  |
| Final polymerization | 72 °C | 05:00 | 1x |

* different annealing temperatures were tested (52 °C – 59 °C) while 55 °C was proved as optimal


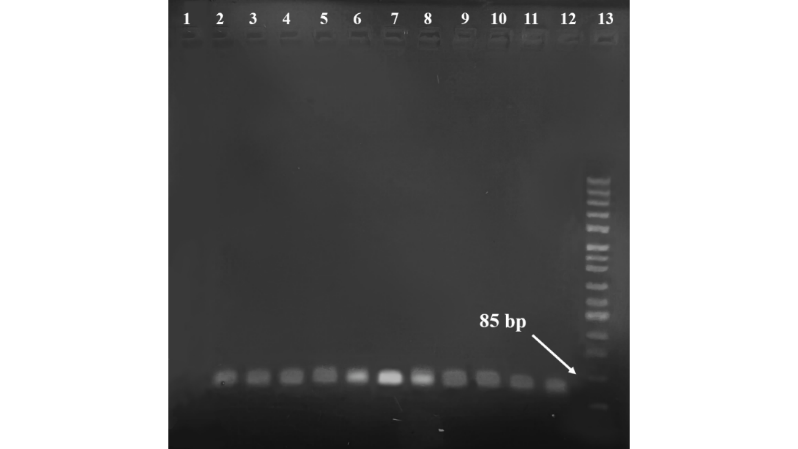
The gel electrophoresis technique in a 2 % agarose gel was utilized to visualize the products created in the PCR. The gel was prepared by boiling 1 g of agarose (Serva, Heidelberg, Germany) in 50 mL of Tris-borate-EDTA buffer (TBE) until complete dissolution. For staining and visualization of nucleic acids, 5 𝜇L of GoodView dye (SBS Genetech CO., Ltd., Beijing, China) was added to the agarose gel. Dissolved gel was evenly poured into the assembled electrophoretic tray. After solidification, 10 𝜇L of the PCR sample, negative control and DNA ladder (Canvax, **Valladolid,** Spain) was pipetted into the wells of the gel. Electrophoresis was performed in the presence of TBE at 100 V for 60 min (PowerPac™, Bio-Rad, Hercules, California). The amplified DNA fragments were visualized under ultraviolet radiation on a UV transilluminator MUV 21-312-220 (Major Science CO., Ltd, Taoyuan City, Taiwan) at a wavelength of 254 nm. Optimal melting temperature of both set of primers was determined by figure S1 and S2.

**Fig. S1:** Gel electrophoresis. Samples on the gel: **1** – *ACT1* negative control; **2** – 52 °C; **3** – 52.2 °C; **4** – 52.7 °C; **5** – 53.4 °C; **6** – 54.2 °C; **7** – 55.1 °C; **8** – 55.9 °C; **9** – 56.8 °C; **10** – 58.3 °C; **11** – 58.8 °C; **12** – 59 °C; **13** – 50 – 1000 bp ladder;


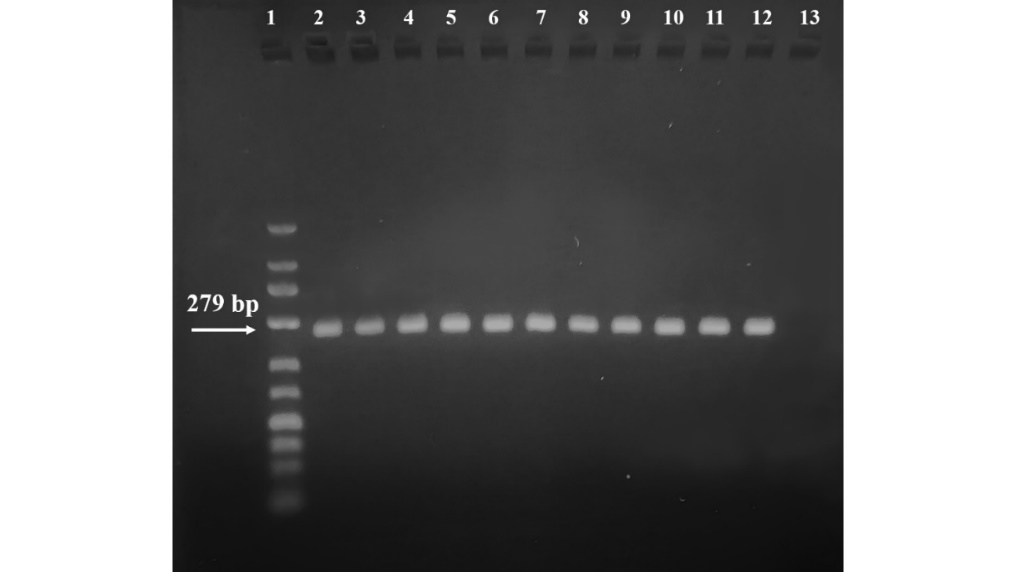


**Fig. S2:** Gel electrophoresis. Samples on the gel: **1** – *nuc* negative control; **2** – 52 °C; **3** – 52.2 °C; **4** – 52.7 °C; **5** – 53.4 °C; **6** – 54.2 °C; **7** – 55.1 °C; **8** – 55.9 °C; **9** – 56.8 °C; **10** – 58.3 °C; **11** – 58.8 °C; **12** – 59 °C; **13** – 25 – 700 bp ladder.


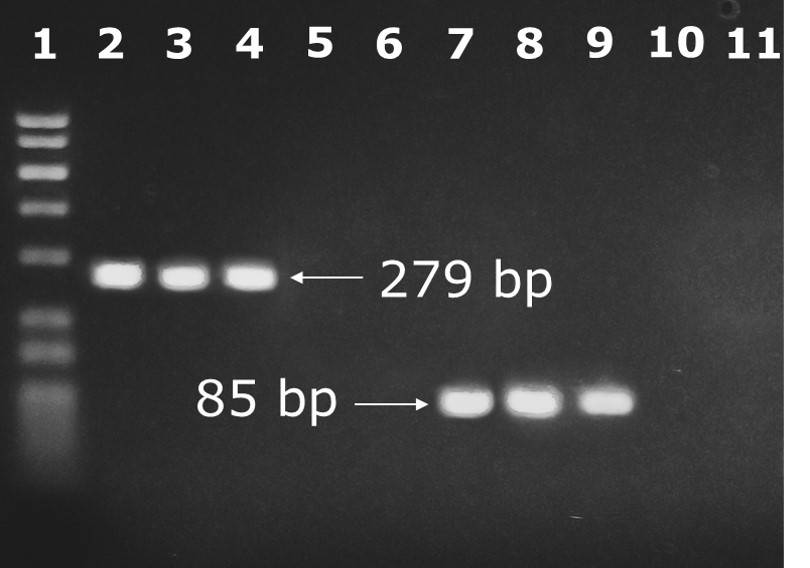
*1.2 Determination of 25 µM PMA concentration by using PCR*

**Fig. S3:** Gel electrophoresis. Samples on the gel: **1** – 25–700 bp ladder; **2** – gDNA of *S. aureus* live cells, without PMA; **3** – gDNA of *S. aureus* live cells, with 25 µM PMA; **4** – gDNA of *S. aureus* dead cells, without PMA; **5** – gDNA of *S. aureus* dead cells, with 25 µM PMA; **6** – *nuc* negative control; **7** – gDNA of *C. albicans* live cells, without PMA; **8** – gDNA of *C. albicans* live cells, with 25 µM PMA; **9** – gDNA of *C. albicans* dead cells, without PMA; **10** – gDNA of *C. albicans* dead cells, with 25 µM PMA; **11** – *ACT1* negative control.

*1.3 Confirmation of amplicons in PMA-qPCR*


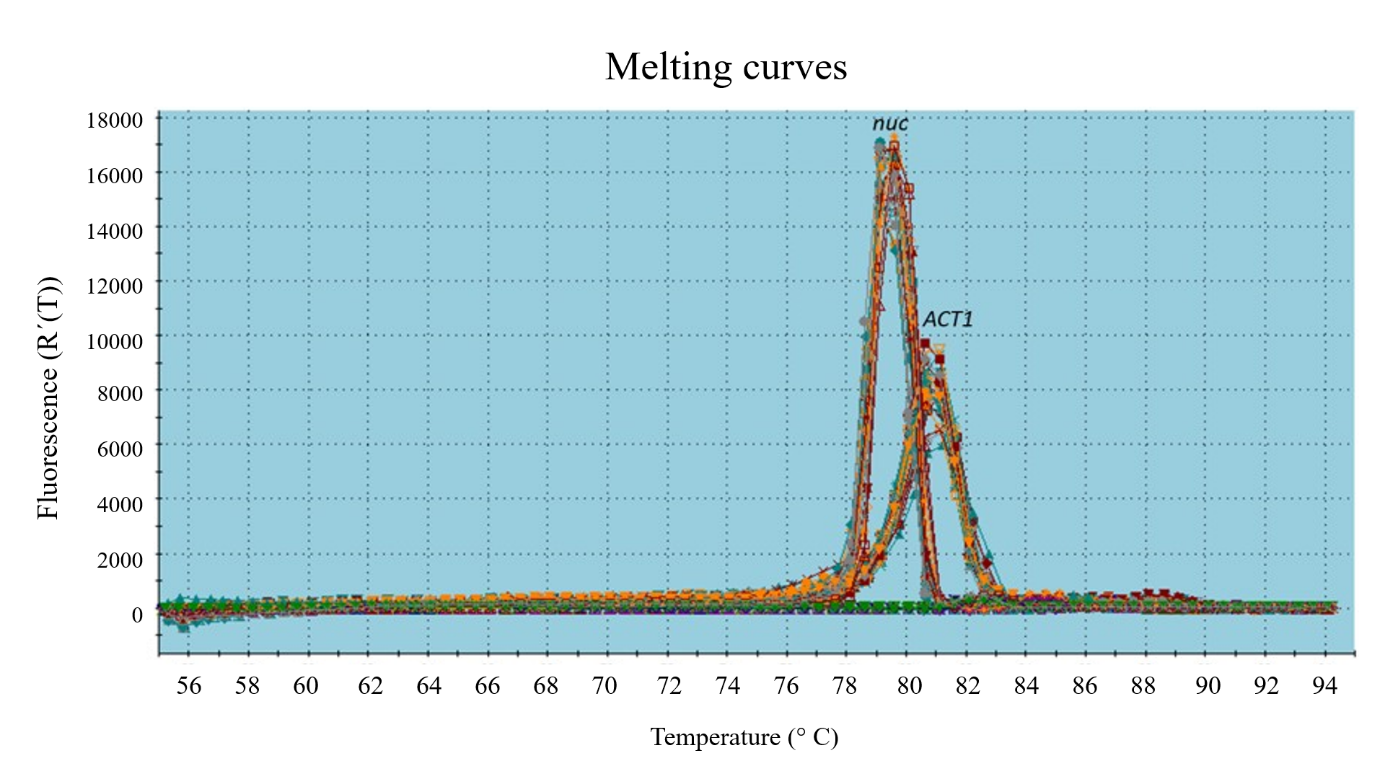
The presence of a specific amplicons in the reaction mixture were confirmed by melting curve analysis with a melting point Tm ranging from 77.3 to 82.8 for *ACT1* and 76.5 to 81.1 for *nuc*, respectively.

**Fig. S4:** Dissociation/melting curves of the bacterial (*nuc*) and yeast (*ACT1*) gene qPCR product.

*1.4 Effectiveness of 50 µM PMA dye*

Figure S5 shows a representative example of qPCR amplification curves after treatment of biofilm cell samples with 50 µM PMA. Apparently, 50 µM PMA represented a very high concentration, affecting DNA amplification of live cells as well, as dCt_live C. albicans_ was 2.94 and dCt_live S. aureus_ was 2.01. The differences in dCt of dead cell controls were also higher than in the case of 25 µM PMA. dCt_dead C. albicans_ was 11.78 and dCt_dead S. aureus_ was 11.41.


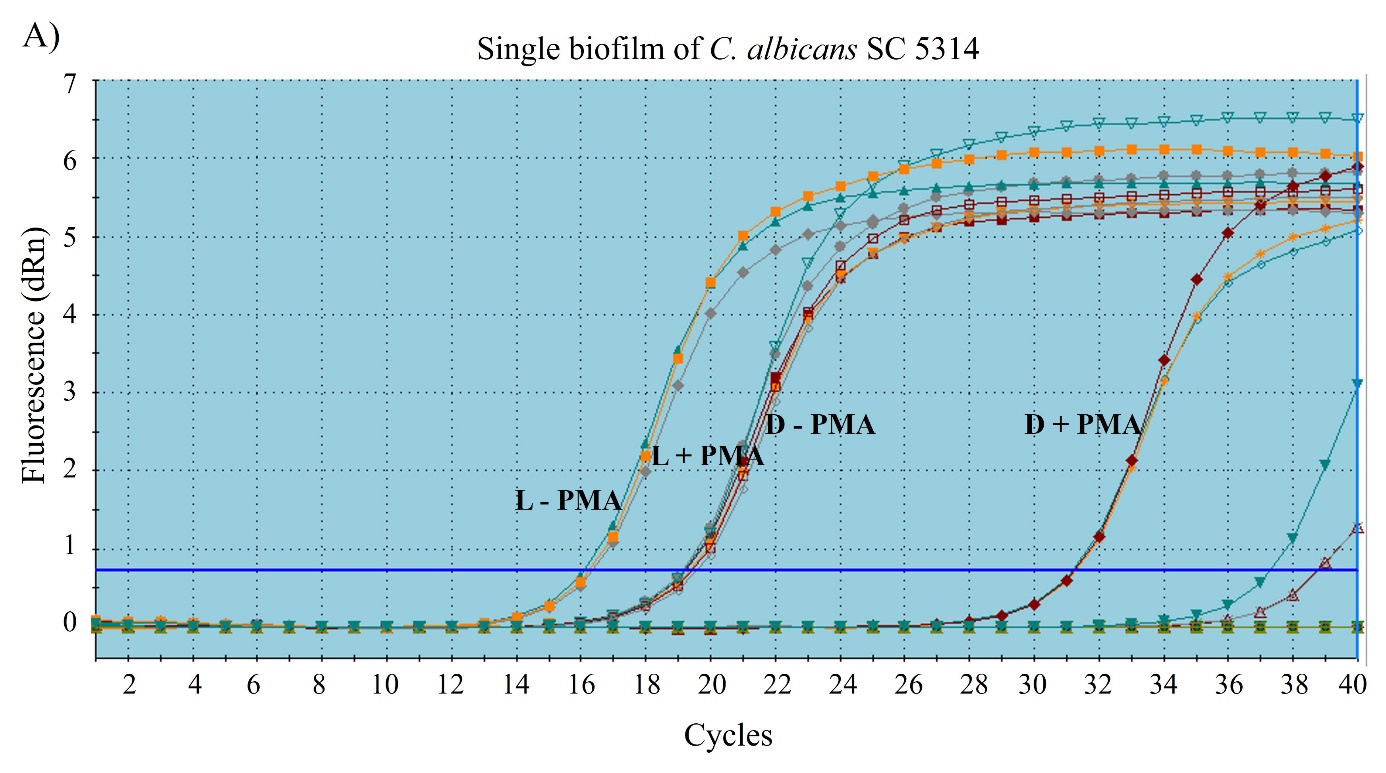


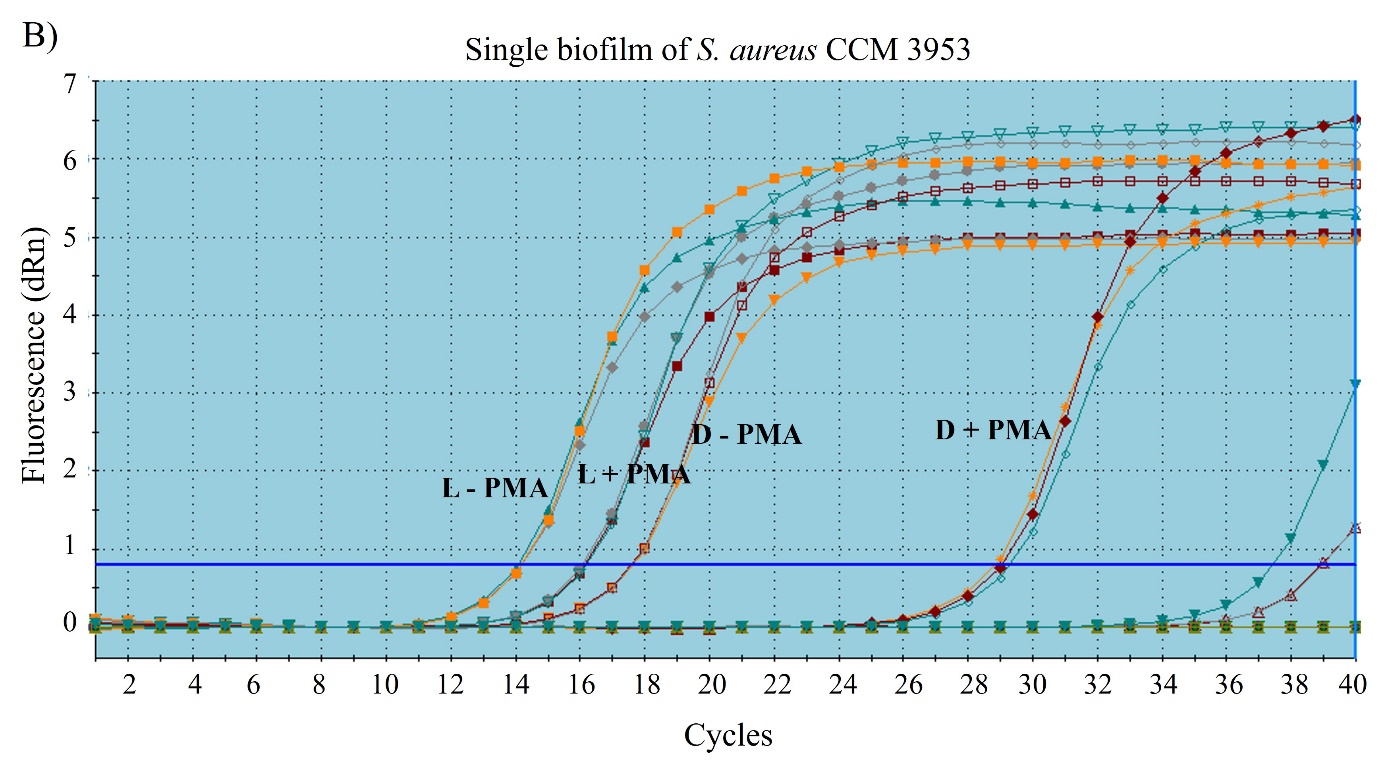


**Fig. S5:** Amplification curves for gDNA of live and dead biofilm cells with and without 50 µM PMA. DNA originated from single biofilm of *C. albicans* (A) and *S. aureus* (B). PMA-qPCR was performed using gDNA of each strain with the respective primers.

L + PMA = live cell control with PMA; L - PMA = live cell control without PMA; D + PMA = dead cell control with PMA; D - PMA = dead cell control without PMA.

1.5 *Effect of irradiation on dual biofilm*

Dual biofilm was done according to the procedure describe in paragraph. 2.2. Biofilm was irradiated for 5 min and then, biofilm cells were scrapped and serially diluted in PBS. Then, samples were plated to Petri dishes, how it is described in paragraph 2.2. Results summarized in Fig.S6 proved that irradiation for 5 min did not have any inhibitory effect on microorganisms in mixed biofilm.


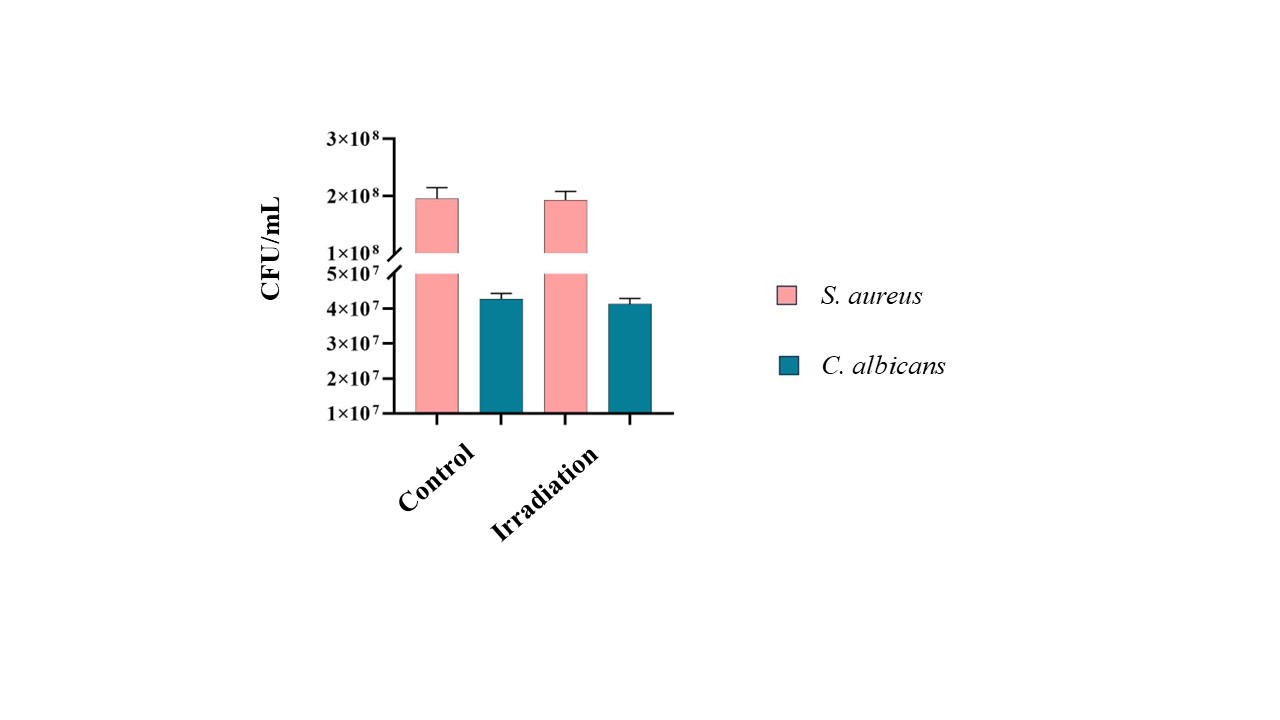
**Fig. S6:** Effect of irradiation (5 min) on dual biofilm of *C. albicans* and *S. aureus*.
