# Supplementary figures and images for: Practical application of PMA–qPCR assay for determination of viable cells of inter-species biofilm of Candida albicans–Staphylococcus aureus
Source: Biol Methods Protoc. 2024 Nov 18;9(1):bpae081. doi: 10.1093/biomethods/bpae081 (PMC11631528; doi:10.1093/biomethods/bpae081)

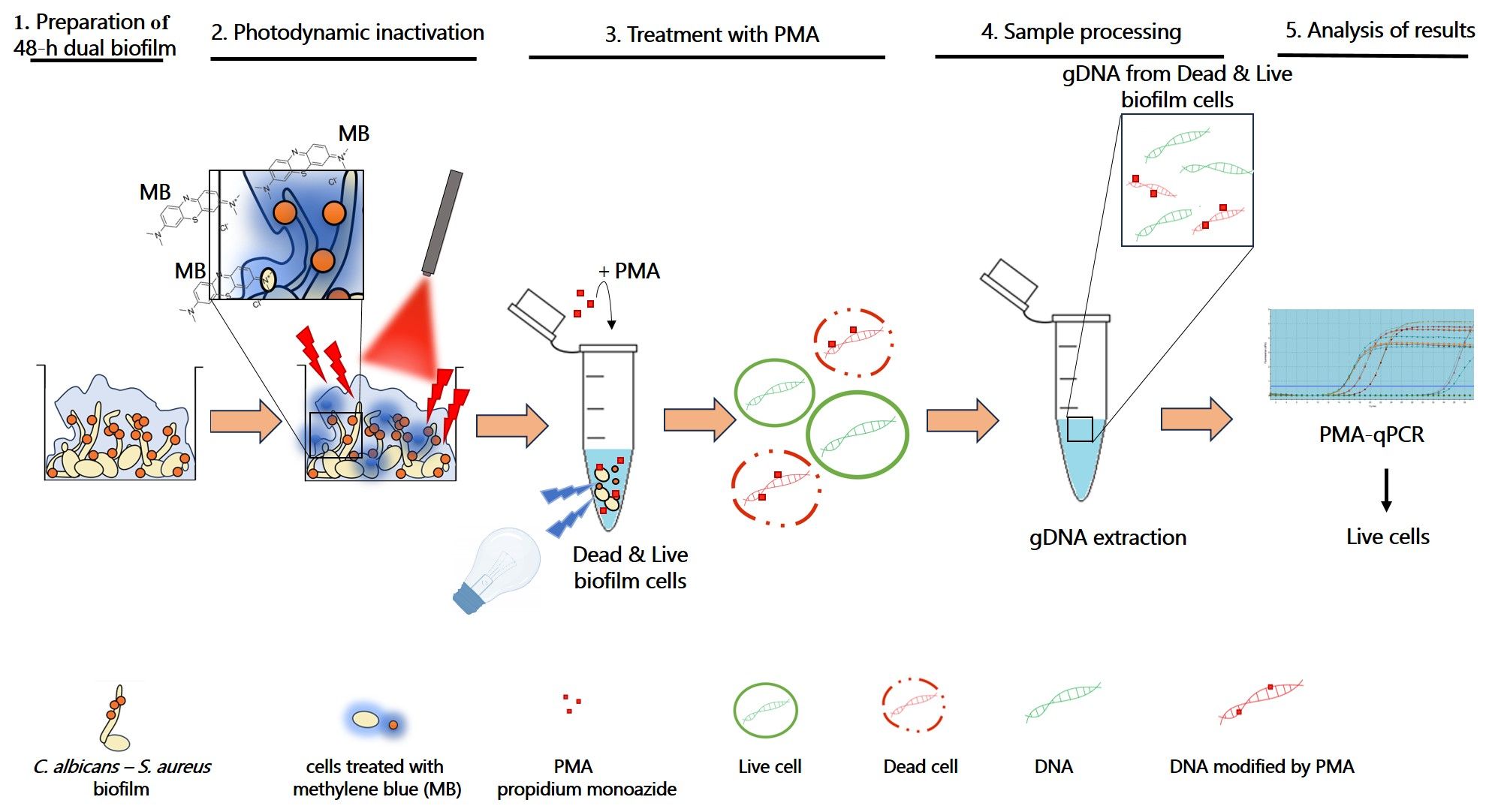

Supplement: bpae081_Supplementary_Data [file bpae081_supplementary_data.zip › Graphical abstract.jpg]
